# Outcomes of Calcium-Channel Blocker Use in Patients With Multiple Myeloma: A Propensity-Matched Study From the Global Federated Health Research Network

**DOI:** 10.7759/cureus.88087

**Published:** 2025-07-16

**Authors:** Sarah Eidbo, John H Malin, Maxim Barnett, Thomas Stavola, Festus Ibe, Lucas Chierici Pereira, Abhishek Shah, Ryan Mayo

**Affiliations:** 1 Internal Medicine, Einstein Medical Center Philadelphia, Philadelphia, USA; 2 Hematology and Oncology, Einstein Medical Center Philadelphia, Philadelphia, ARE

**Keywords:** calcium-channel blockers, database, multiple myeloma, outcomes, retrospective analysis

## Abstract

Introduction: Multiple myeloma (MM) is a plasma cell hyperproliferative disorder resulting in a classic presentation including anemia, renal dysfunction, bone pain, and hypercalcemia. Many patients with MM also develop unrelated comorbid conditions, such as hypertension or congestive heart failure, that warrant the prescription of calcium-channel blockers (CCBs). Little is known about how MM-associated hypercalcemia could be impacted by CCB use. This study aimed to determine how CCBs could impact outcomes in patients with MM.

Methods: Utilizing TriNetX’s US Collaborative Network and ICD-10 codes, we identified adult patients with MM prescribed CCBs and compared them to patients with MM not prescribed CCBs. Cohorts were propensity-matched according to age, sex, race, and comorbidities. We followed these cohorts for five years to assess rates of acute kidney injury (AKI), dialysis initiation (hemodialysis (HD)), seizures, atrial fibrillation/atrial flutter (AF/AFL), ventricular fibrillation/ventricular tachycardia (VF/VT), ICU admission, and all-cause mortality.

Results: Cohorts were propensity-matched with each CCB cohort compared with a group that was not prescribed any CCB. Compared to any CCBs, the MM no CCB cohort had lower rates of AKI (hazard ratio (HR) 0.937, 95% CI 0.911 - 0.964, p<0.0001), HD (HR 0.587, 95% CI 0.551 - 0.625, p<0.0001), seizures (HR 0.823, 95% CI 0.755 - 0.898, p=0.0002), AF/AFL (HR 0.942, 95% CI 0.905 - 0.98, p<0.0001), and VF/VT (HR 0.981, 95% CI 0.901 - 1.076, p<0.0001). The MM no CCB cohort had higher rates of mortality (HR 1.031, 95% CI 1.004 - 1.059, p<0.0001).

The MM no amlodipine cohort had lower rates of AKI (HR 1.011, 95% CI 0.98 - 1.044, p<0.0001), HD (HR 0.749, 95% CI 0.698 - 0.803, p<0.0001), seizures (HR 1.034, 95% CI 0.939 - 1.139, p<0.0001), and ICU admission (HR 0.159, 95% CI 0.118 - 0.214, p=0.0367) compared to the amlodipine cohort. The MM no amlodipine cohort had increased risk of AF/AFL (HR 1.44, 95% CI 1.371 - 1.513, p<0.0001), VF/VT (HR 1.464, 95% CI 1.322 - 1.62, p<0.0001), and mortality (HR 1.09, 95% CI 1.058 - 1.123, p<0.0001).

The MM no nicardipine cohort had reduced risk of mortality (HR 0.754, 95% CI 0.633 - 0.899, p=0.0002) compared to the nicardipine cohort.

The MM no nifedipine cohort had less risk of HD (HR 0.507, 95% CI 0.402 - 0.639, p=0.0057) and mortality (HR 0.935, 95% CI 0.839 - 1.043, p=0.0395) compared to the nifedipine cohort. The MM no nifedipine cohort had a higher risk of AF/AFL (HR 1.59, 95% CI 1.306 - 1.935, p=0.0003) and VF/VT (HR 1.927, 95% CI 1.326 - 2.799, p=0.0012).

The MM no diltiazem cohort had lower rates of HD (HR 0.941, 95% CI 0.79 - 1.121, p=0.0256), AF/AFL (HR 0.322, 95% CI 0.293 - 0.353, p<0.0001), and mortality (HR 0.775, 95% CI 0.728 - 0.825, p=0.0006) than the diltiazem cohort. The MM no diltiazem cohort demonstrated higher rates of AKI (HR 1.086, 95% CI 1.005-1.174, p=0.0003).

The MM no verapamil cohort had a reduced risk of AF/AFL (HR 0.949, 95% CI 0.798-1.129, p=0.0011) compared to the verapamil cohort. The MM no verapamil cohort had a higher risk of AKI (HR 1.369, 95% CI 1.203 - 1.557, p=0.0001) and HD (HR 1.329, 95% CI 0.985 - 1.795, p=0.0392).

Conclusions: Even after risk stratification and propensity-score matching, there appear to be differences in outcomes between patients with MM on various CCBs. The hypercalcemia in MM may be perpetuated by CCBs, leading to poor outcomes in certain patients. These differences warrant further exploration.

## Introduction

Multiple myeloma, or MM, is a hyperproliferative disorder of plasma cells that can cause anemia, renal dysfunction, bone pain, and hypercalcemia through excessive monoclonal immunoglobulin production that leads to end-organ damage ​​​​[1-3]. MM is thought to start as a monoclonal gammopathy of undetermined significance (MGUS), a benign condition characterized by excess monoclonal immunoglobulins but no end-organ damage [4, 5]. Approximately 1% of MGUS progresses to MM in one year, though the cause of this progression is uncertain [4, 5]. Multiple oncogenes have been implicated, including NRAS, BRAF, and KRAS [6, 7]. Despite the underlying cause, the hyperviscosity caused by excess immunoglobulins can lead to renal tubular damage and neurologic derangements [8]. Clonal proliferation within the bone marrow can induce pancytopenia by crowding out healthy marrow [8, 9]. Involved bone marrow stroma is also theorized to overexpress receptor activator of nuclear factor kappa-B ligand (RANKL), leading to osteoclast activation and bone resorption [8, 9]. By activating osteoclasts, MM can thus also manifest with hypercalcemia and lytic bone lesions [8, 9].

As MM is most commonly a geriatric condition, it is important to consider comorbid conditions that may impact outcomes in this unique patient population [2, 10]. Calcium-channel blockers (CCBs) are common medications used to manage a variety of cardiovascular conditions, including hypertension, systolic and diastolic heart failure, chronic stable angina, and coronary artery disease [11]. These conditions are not exclusive to geriatric patients but do occur more frequently in the elderly [2, 11]. Given that patients with MM will likely be diagnosed with a comorbid cardiovascular condition requiring CCBs for management, it is important to understand their impact. There is little available data on how CCBs impact MM and its associated hypercalcemia. This retrospective analysis aimed to determine how CCBs could affect outcomes for patients diagnosed with MM. 

To more thoroughly understand the effects of CCBs on MM, it is helpful to categorize them. CCBs are broken into two groups: dihydropyridines and non-dihydropyridines [11]. Though both prevent intracellular movement of calcium by inhibiting L-type voltage-gated calcium channels, dihydropyridines primarily function peripherally as vasodilators [12-14]. Non-dihydropyridines inhibit sinoatrial (SA) and atrioventricular (AV) nodes, slowing cardiac conduction and reducing contractility [12-14]. Dihydropyridine CCBs in this study included amlodipine, nicardipine, and nifedipine. Non-dihydropyridine CCBs included diltiazem and verapamil. 

## Materials and methods

Eligibility

The TriNetX US Collaborative Network, a nationwide de-identified health information database, was utilized for this study to extract data across multiple healthcare organizations (HCOs) according to International Classification of Diseases (ICD-10) codes [15]. ICD-10 codes used included those related to MM and one of five CCBs, including amlodipine, diltiazem, nicardipine, nifedipine, and verapamil. ICD-10 codes were also utilized to define outcomes, including acute kidney injury (AKI), dialysis initiation (hemodialysis (HD)), seizures, atrial fibrillation/atrial flutter (AF/AFL), ventricular fibrillation/ventricular tachycardia (VF/VT), ICU admission, and all-cause mortality. ICD-10 codes used to identify MM are listed in Table 1. ICD-10 codes used to identify CCBs studied are listed in Table 2. ICD-10 codes used to identify outcomes are listed in Table 3. 

**Table 1:** Multiple myeloma ICD-10 codes International Classification of Diseases (ICD-10) codes utilized to identify patients with multiple myeloma [15].

Condition	ICD-10 code
Multiple myeloma	UMLS:ICD10CM:C90.02: Multiple myeloma in relapse
	UMLS:ICD10CM:C90.0: Multiple myeloma
	UMLS:ICD10CM:C90.00: Multiple myeloma not having achieved remission
	UMLS:ICDO3:9732/3: Plasma cell myeloma

**Table 2:** CCB ICD-10 codes International Classification of Diseases (ICD-10) codes utilized to identify calcium channel blockers (CCBs) [15].

CCB	ICD-10 code
Amlodipine	NLM:RXNORM:17767: Amlodipine
Diltiazem	NLM:RXNORM:3443: Diltiazem
	NLM:RXNORM:314592: Diltiazem maleate
Nicardipine	NLM:RXNORM:7396: Nicardipine
Nifedipine	NLM:RXNORM:7417: Nifedipine
Verapamil	NLM:RXNORM:11170: Verapamil
Any CCB	All above ICD-10 codes, plus:
	NLM:ATC:C08C: Selective calcium channel blockers with mainly vascular effects
	NLM:VA:CV200: Calcium channel blockers
	NLM:ATC:C08E: Non-selective calcium channel blockers
	NLM:ATC:C08D: Selective calcium channel blockers with direct cardiac effects
	NLM:ATC:C08: Calcium channel blockers
	NLM:ATC:C08EX: Other non-selective calcium channel blockers (deprecated 2020)

**Table 3:** Outcomes identified by ICD-10 codes International Classification of Diseases (ICD-10) codes used to identify outcomes, including acute kidney injury (AKI) or failure, initiation of dialysis (hemodialysis (HD)), seizures, atrial fibrillation or atrial flutter (AF/AFL), ventricular fibrillation or ventricular tachycardia (VF/VT), intensive care unit admission (ICU), and all-cause mortality [15].

Outcome	ICD-10 code
AKI	UMLS:ICD10CM:N17: Acute kidney failure
	UMLS:ICD10CM:N17.9: Acute kidney failure, unspecified
	UMLS:ICD10CM:N17.8: Other acute kidney failure
	UMLS:ICD10CM:N17.1: Acute kidney failure with acute cortical necrosis
	UMLS:ICD10CM:N17.0: Acute kidney failure with tubular necrosis
	UMLS:ICD10CM:N17.2: Acute kidney failure with medullary necrosis
	UMLS:ICD10CM:N17-N19: Acute kidney failure and chronic kidney disease
HD	UMLS:CPT:90945: Dialysis procedure other than hemodialysis (eg, peritoneal dialysis, hemofiltration, or other continuous renal replacement therapies), with single evaluation by a physician or other qualified health care professional
	UMLS:CPT:90947: Dialysis procedure other than hemodialysis (eg, peritoneal dialysis, hemofiltration, or other continuous renal replacement therapies) requiring repeated evaluations by a physician or other qualified health care professional, with or without substantial revision of dialysis prescription
	UMLS:SNOMED:108241001: Dialysis procedure
	UMLS:CPT:1006747: Hemodialysis access, Intervascular cannulation for extracorporeal circulation, or shunt insertion procedures on arteries and veins
	UMLS:SNOMED:302497006: Hemodialysis
	UMLS:ICD9CM:39.95: Hemodialysis
	UMLS:CPT:1012752: Hemodialysis procedure
	UMLS:CPT:90935: Hemodialysis procedure with single evaluation by a physician or other qualified health care professional
	UMLS:CPT:1006747: Hemodialysis access, Intervascular cannulation for extracorporeal circulation, or shunt insertion procedures on arteries and veins
	UMLS:CPT:90937: Hemodialysis procedure requiring repeated evaluation(s) with or without substantial revision of dialysis prescription
	UMLS:ICD10CM:Z99.2: Dependence on renal dialysis
Seizures	UMLS:ICD10CM:R56: Convulsions, not elsewhere classified
	UMLS:ICD10CM:G40.89: Other seizures
AF/AFL	UMLS:ICD10CM:I48.91: Unspecified atrial fibrillation
	UMLS:ICD10CM:I48.0: Paroxysmal atrial fibrillation
	UMLS:ICD10CM:I48.1: Persistent atrial fibrillation
	UMLS:ICD10CM:I48.2: Chronic atrial fibrillation
	UMLS:ICD10CM:I48.19: Other persistent atrial fibrillation
	UMLS:ICD10CM:I48.21: Permanent atrial fibrillation
	UMLS:ICD10CM:I48: Atrial fibrillation and flutter
	UMLS:ICD10CM:I48.20: Chronic atrial fibrillation, unspecified
	UMLS:ICD10CM:I48.11: Longstanding persistent atrial fibrillation
	UMLS:ICD10CM:I48.92: Unspecified atrial flutter
	UMLS:ICD10CM:I48.3: Typical atrial flutter
	UMLS:ICD10CM:I48.4: Atypical atrial flutter
	UMLS:ICD10CM:I48.9: Unspecified atrial fibrillation and atrial flutter
VF/VT	UMLS:ICD10CM:I49.01: Ventricular fibrillation
	UMLS:ICD10CM:I49.0: Ventricular fibrillation and flutter
	UMLS:ICD10CM:I47.2: Ventricular tachycardia
	UMLS:ICD10CM:I47.20: Ventricular tachycardia, unspecified
	UMLS:ICD10CM:I47.29: Other ventricular tachycardia
ICU Admission	UMLS:SNOMED:305351004: Admission to intensive care unit
Mortality	Deceased: Deceased

Patients included in the study were adults 18 years or older with an MM diagnosis, with or without prescribed CCBs as listed above. The index event, defined as the day the patient first met criteria for a cohort, was set as the diagnosis of MM. Patients with outcomes as listed above that occurred prior to the index event were excluded from the study. 

Patients were divided into multiple cohorts: a cohort with MM on no CCBs, a cohort with MM on amlodipine, a cohort with MM on diltiazem, MM on nicardipine, MM on nifedipine, and MM on verapamil. These cohorts were propensity-matched prior to comparisons according to age at index event, sex, race, ethnicity, and comorbid conditions as listed in Table 4. 

**Table 4:** Propensity-score matching ICD-10 codes International Classification of Diseases (ICD-10) codes utilized to identify demographics and conditions used for cohort propensity-score matching [15]. Racial information is reported as documented in the source data.

Demographic	ICD-10 code
Age at index	AI
Female	F
Black or African American race	2054-5
Male	M
White or Caucasian race	2106-3
American Indian or Alaska Native race	1002-5
Unknown race	UNK
Native Hawaiian or Other Pacific Islander race	2076-8
Unknown ethnicity	UN
Non-Hispanic or Latino race	2186-5
Hispanic or Latino race	2135-2
Other race	2131-1
Asian race	2028-9
Neoplasms	C00-D49
Diseases of the respiratory system	J00-J99
Diseases of the nervous system	G00-G99
Diseases of the digestive system	K00-K95
Diseases of the circulatory system	I00-I99

Statistical analysis

To analyze results, index events and time windows were defined. The index event was defined as the day of MM diagnosis. The time window was defined as the period of time after the index event during which an outcome could occur. This study used a time window of five years. 

Kaplan-Meier analyses were utilized for survival analysis with log-rank testing, hazard ratios (HRs), and proportionality testing performed alongside median survival testing and survival probability as well. Patients who exited a cohort prior to the end of the time window were censored from analysis. 

## Results

MM and any CCB

Propensity-score matching revealed 43,555 patients in each cohort-MM with and without any CCBs. The average age at index for the MM no CCB cohort was 68.1 +/- 12.3 years, compared to the CCB cohort at 68.0 +/- 11.7 years. The no CCB cohort was approximately 43.7% (n=19,034) female compared to the CCB cohort at 43.96% (n=19,132) female. The no CCB cohort predominantly consisted of Caucasian (58.84%, n=25,629), followed by African American (17.61%, n=7,668), Hispanic/Latino (4.18%, n=1,821), and Asian (3.49%, n=1,521) patients. The CCB cohort predominantly had Caucasian (58.32%, n=25,399), followed by African American (17.44%, n=7,596), Hispanic/Latino (4.45%, n=1,942), and Asian (3.92%, n=1,708) patients. The MM no CCB cohort demonstrated significantly lower rates of AKI (HR 0.937, 95% CI 0.911 - 0.964, p<0.0001), HD (HR 0.587, 95% CI 0.551 - 0.625, p<0.0001), seizures (HR 0.823, 95% CI 0.755 - 0.898, p=0.0002), AF/AFL (HR 0.942, 95% CI 0.905 - 0.98, p<0.0001), and VF/VT (HR 0.981, 95% CI 0.901 - 1.076, p<0.0001). The MM no CCB cohort demonstrated significantly higher rates of all-cause mortality (HR 1.031, 95% CI 1.004-1.059, p<0.0001). There was no significant difference between cohorts in rates of ICU admission (HR 0.179, 95% CI 0.137 - 0.234, p=0.3441). Results are displayed in Table 5. 

**Table 5:** Rates of adverse outcomes in patients with MM on CCBs Outcomes after propensity-score matching for each comparison: multiple myeloma (MM) without calcium-channel blocker (CCB) compared to each CCB as listed. CCBs are grouped according to the dihydropyridine or non-dihydropyridine class. Statistical significance is defined as a p-value of or less than p=0.05. Cells with an asterisk (*) indicate a reduced risk of this outcome in the cohort without calcium-channel blockers. Cells with (+) indicate an increased risk of this outcome in the cohort without calcium-channel blockers. Cells with (-) indicate that the value could not be calculated, as no patients of either cohort met the criteria. AKI: acute kidney injury; HD: hemodialysis; AF/AFL: atrial fibrillation/atrial flutter; VT/VT: ventricular fibrillation/ventricular tachycardia

P-value	CCB	Group 1	Group 2	AKI	HD	Seizures	AF/AFL	VF/VT	ICU	Mortality
	Any	MM no CCB	MM any CCB	0.0001*	0.0001*	0.0002*	0.0001*	0.0001*	0.3441	0.0001+
	Dihydropyridines	MM no CCB	MM and amlodipine	0.0001*	0.0001*	0.0001*	0.0001+	0.0001+	0.0367*	0.0001+
		MM no CCB	MM and nicardipine	0.357	0.094	0.1651	0.2719	0.7308	-	0.0002*
		MM no CCB	MM and nifedipine	0.6626	0.0057*	0.2586	0.0003+	0.0012+	0.6554	0.0395*
	Non-dihydropyridines	MM no CCB	MM and diltiazem	0.0003+	0.0256*	0.0535	0.0001*	0.2471	0.2151	0.0006*
		MM no CCB	MM and verapamil	0.0001+	0.0392+	0.6678	0.0011*	0.1727	-	0.353
HR	CCB	Group 1	Group 2	AKI	HD	Seizures	AF/AFL	VF/VT	ICU	Mortality
	Any	MM no CCB	MM any CCB	0.937*	0.587*	0.823*	0.942*	0.981*	0.179	1.031+
	Dihydropyridines	MM no CCB	MM and amlodipine	1.011*	0.749*	1.034*	1.44+	1.464+	0.159*	1.09+
		MM no CCB	MM and nicardipine	1.218	1.371	0.365	0.964	0.596	-	0.754*
		MM no CCB	MM and nifedipine	0.922	0.507*	0.865	1.59+	1.927+	0.204	0.935*
	Non-dihydropyridines	MM no CCB	MM and diltiazem	1.086+	0.941*	0.943	0.322*	0.837	2.471	0.775*
		MM no CCB	MM and verapamil	1.369+	1.329+	1.035	0.949*	0.666	-	0.98
95% CIs	CCB	Group 1	Group 2	AKI	HD	Seizures	AF/AFL	VF/VT	ICU	Mortality
	Any	MM no CCB	MM any CCB	0.551 - 0.625*	0.551 - 0.625*	0.755 - 0.8988*	0.905 - 0.98*	0.901 - 1.076*	0.137 - 0.234	1.004 - 1.059+
	Dihydropyridines	MM no CCB	MM and amlodipine	0.98 - 1.044*	0.698 - 0.803*	0.939 - 1.139*	1.371 - 1.513+	1.322 - 1.62+	0.118 - 0.214*	1.058 - 1.123+
		MM no CCB	MM and nicardipine	0.969 - 1.533	0.829 - 2.268	0.235 - 0.568	0.693 - 1.431	0.351 - 1.015	-	0.633 - 0.899*
		MM no CCB	MM and nifedipine	0.817 - 1.04	0.402 - 0.639*	0.625 - 1.196	1.306 - 1.905+	1.326 - 2.799+	0.07 - 0.596	0.839 - 1.043*
	Non-dihydropyridines	MM no CCB	MM and diltiazem	1.005 - 1.174+	0.79 - 1.121*	0.745 - 1.195	0.293 - 0.353*	0.684 - 1.023	0.479 - 12.738	0.728 - 0.825*
		MM no CCB	MM and verapamil	1.203 - 1.557+	0.985 - 1.795+	0.733 - 1.461	0.798 - 1.129*	0.5 - 0.887	-	0.879 - 1.093

Dihydropyridines

MM and Amlodipine

Propensity-score matching revealed 35,452 patients in each cohort, MM with and without amlodipine. The MM no amlodipine cohort was 68.4 +/- 11.9 years at index, compared to the amlodipine cohort at 68.3 +/- 11.6 years. The MM no amlodipine cohort was 43.96% (n=15,586) female, compared to the amlodipine cohort at 44.8% (n=15,888) female. The MM no amlodipine cohort predominantly consisted of Caucasian (56.62%, n=20,073), followed by African American (20.9%, n=7,410), Hispanic/Latino (4.21%, n=1,494), and Asian (3.39%, n=1,202) patients. The amlodipine cohort similarly predominantly had Caucasian (55.85%, n=19,801), followed by African American (20.54%, n=7,282), Hispanic/Latino (4.67%, n=1,655), and Asian (3.7%, n=1,331) patients. The MM no amlodipine cohort demonstrated significantly lower rates of AKI (HR 1.011, 95% CI 0.98 - 1.044, p<0.0001), HD (HR 0.749, 95% CI 0.698 - 0.803, p<0.0001), seizures (HR 1.034, 95% CI 0.939 - 1.139, p<0.0001), and ICU admission (HR 0.159, 95% CI 0.118 - 0.214, p=0.0367). The MM no amlodipine cohort demonstrated significantly increased risk of AF/AFL (HR 1.44, 95% CI 1.371 - 1.513, p<0.0001), VF/VT (HR 1.464, 95% CI 1.322 - 1.62, p<0.0001), and all-cause mortality (HR 1.09, 95% CI 1.058 - 1.123, p<0.0001). Results are displayed in Table 5. 

MM and Nicardipine

Propensity-score matching identified 792 patients in each cohort, with and without nicardipine. The MM no nicardipine cohort was 69.1 +/- 11.4 years at index event, compared to the nicardipine cohort at 68.8 +/- 11.6 years. The MM no nicardipine cohort and nicardipine cohorts were approximately 38.01% (n=301 per cohort) female. The MM no nicardipine cohort predominantly consisted of Caucasian (72.48%, n=574), followed by African American (14.52%, n=115), Hispanic/Latino (4.55%, n=36), and Asian (4.17%, n=33) patients. The nicardipine cohort predominantly had Caucasian (70.2%, n=556), followed by African American (14.02%, n=111), Hispanic/Latino (5.05%, n=40), and Asian (4.98%, n=38) patients. The MM no nicardipine cohort demonstrated significantly reduced risk of all-cause mortality (HR 0.754, 95% CI 0.633 - 0.899, p=0.0002). The cohorts demonstrated no significant difference between rates of AKI (HR 1.218, 95% CI 0.969 - 1.533, p=0.357), HD (HR 1.371, 95% CI 0.829 - 2.268, p=0.094), seizures (HR 0.365, 95% CI 0.235 - 0.568, p=0.1651), AF/AFL (HR 0.964, 95% CI 0.693 - 1.341, p=0.2719), VT/VF (HR 0.596, 95% CI 0.351 - 1.015, p=0.7308), or ICU admission (no patients in either cohort were recorded with this outcome. Results are displayed in Table 5. 

MM and Nifedipine

After propensity-score matching, 2,666 patients were identified in each cohort. The MM no nifedipine cohort was 66.4 +/- 12.7 years at index, compared to 66.5 +/- 12.9 years at index for the nifedipine cohort. The MM no nifedipine cohort was 46.36% (n=1,236) female compared to the nifedipine cohort at 47.19% (n=1,258) female. The MM no nifedipine cohort predominantly had Caucasian (44.07%, n=1,175), followed by African American (32.03%, n=854), Hispanic/Latino (5.36%, n=143), and Asian (3.08%, n=82) patients. The nifedipine cohort similarly mostly comprised Caucasian (43.92%, n=1,171), followed by African American (31.43%, n=838), Hispanic/Latino (6.04%, n=161), and Asian (2.96%, n=79) patients. The MM no nifedipine cohort demonstrated significantly reduced risk of HD (HR 0.507, 95% CI 0.402 - 0.639, p=0.0057), and all-cause mortality (HR 0.935, 95% CI 0.839 - 1.043, p=0.0395). The MM no nifedipine cohort demonstrated significantly higher risk of AF/AFL (HR 1.59, 95% CI 1.306 - 1.935, p=0.0003) and VF/VT (HR 1.927, 95% CI 1.326 - 2.799, p=0.0012). The cohorts demonstrated no difference in rates of AKI (HR 0.922, 95% CI 0.817 - 1.04, p=0.6626), seizures (HR 0.865, 95% CI 0.625 - 1.196, p=0.2586), or ICU admission (HR 0.204, 95% CI 0.07 - 0.596, p=0.6554). Results are displayed in Table 5. 

Non-dihydropyridines 

MM and Diltiazem

After propensity-score matching, 5,729 patients were identified in each cohort, with and without diltiazem. The MM no diltiazem cohort was 71 +/- 11 years at the index event, compared to the diltiazem cohort at 71.3 +/- 10.6 years. The MM no diltiazem cohort was 41.81% (n=2,395) female compared to the diltiazem cohort at 43.01% (n=2,464) female. The diltiazem cohort predominantly had Caucasian (75.86%, n=4,346), followed by African American (10.93%, n=626), Asian (2.51%, n=144), and Hispanic/Latino (2.32%, n=133) patients. The MM no diltiazem cohort also predominantly consisted of Caucasian (74.32%, n=4,206), followed by African American (11.22%, n=643), Asian (3.28%, n=188), and Hispanic/Latino (2.48%, n=142) patients. The MM no diltiazem cohort demonstrated significantly lower rates of HD (HR 0.941, 95% CI 0.79-1.121, p=0.0256), AF/AFL (HR 0.322, 95% CI 0.293-0.353, p<0.0001), and all-cause mortality (HR 0.775, 95% CI 0.728-0.825, p=0.0006). The MM no diltiazem cohort demonstrated significantly higher rates of AKI (HR 1.086, 95% CI 1.005-1.174, p=0.0003). There was no significant difference between cohorts in rates of seizures (HR 0.943, 95% CI 0.745-1.195, p=0.0535), VF/VT (HR 0.837, 95% CI 0.684-1.023, p=0.2471), or ICU admission (HR 2.471, 95% CI 0.479-12.738, p=0.2151). Results are displayed in Table 5. 

MM and Verapamil

Propensity-score matching revealed 2,375 patients per cohort. Both cohorts were 68.5 +/- 11.1 years at index. The MM no verapamil cohort was 41.22% (n=979) female, compared to the verapamil cohort at 41.35% (n=982) female. The MM no verapamil cohort predominantly had Caucasian (71.71%, n=1,703), followed by African American (16.67%, n=396), Hispanic/Latino (2.70%, n=64), and Asian (2.15%, n=51) patients. The verapamil cohort also had predominantly Caucasian (71.12%, n=1,689), followed by African American (16.63%, n=395), Hispanic/Latino (3.24%, n=77), and Asian (1.94%, n=46) patients. The MM no verapamil cohort demonstrated a significantly reduced risk of AF/AFL (HR 0.949, 95% CI 0.798 - 1.129, p=0.0011). The MM no verapamil cohort demonstrated a significantly increased risk of AKI (HR 1.369, 95% CI 1.203 - 1.557, p=0.0001) and HD (HR 1.329, 95% CI 0.985 - 1.795, p=0.0392). There were no significant differences between cohorts in rates of seizures (HR 1.035, 95% CI 0.733 - 1.461, p=0.6678), VF/VT (HR 0.666, 95% CI 0.5 - 0.887, p=0.1727), or all-cause mortality (HR 0.98, 95% CI 0.879 - 1.093, p=0.353). No patients of either cohort were reported to be admitted to the ICU during the time window studied. Results are displayed in Table 5. 

## Discussion

Findings 

Here, we described a retrospective analysis of nationwide data on patients with MM prescribed CCBs compared to those not prescribed CCBs. CCBs are common medications prescribed for a variety of conditions, including but not limited to hypertension, systolic and diastolic heart failure, chronic stable angina, and coronary artery disease [11]. As hypercalcemia is a key feature of MM, understanding how common medications such as CCBs could affect this electrolyte disturbance is of vital importance [8, 9]. To most accurately capture the effects of CCBs on patients with MM, CCBs were broken into dihydropyridines and non-dihydropyridines to explore any difference that could exist via mechanism. Outcomes were also studied for MM with any form of CCB, regardless of mechanism. 

MM with Any CCB

Among the MM with any CCB, there were multiple significant findings worth noting. The cohort without CCBs demonstrated a significant reduction in rates of AKI, HD, AF/AFL, VF/VT, and seizures. As the CCBs studied act by slowing calcium reentry into cardiac cells, it is possible that this inhibition, coupled with hypercalcemia in MM, could lead to increased rates of cardiac arrhythmias [16]. The hypercalcemia in MM has been implicated in the literature for causing cardiac arrhythmias when severe, via shortened QT intervals [16-18]. If the hypercalcemia caused by MM can induce these rhythms alone, then coupling with CCBs could certainly increase the risk. 

CCBs appeared to increase the risk of developing AKIs and the need for HD as well. These medications are typically not associated with AKIs and HD when used in healthy patients [16]. However, MM-induced hypercalcemia can lead to AKI via vasoconstriction, intratubular calcium deposition, and decreased glomerular filtration rates [17, 18]. Despite the vasodilation that CCBs are typically associated with, it appears that patients with MM taking CCBs still have a higher rate of AKI and HD. AKIs are also commonly seen in MM due to the excess free light chains produced that bind Tamm-Horsfall proteins in the distal tubules to form obstructive casts [17, 18]. It is possible that the combination of CCBs and MM-induced hypercalcemia could lead to AKIs and further HD initiation via a synergistic effect of both glomerular vasoconstriction and intratubular calcium deposition. This relationship should be further explored. 

The cohort without CCBs also demonstrated a significantly lower risk of seizures. It is possible that the hypercalcemia induced by MM could be compounded by CCBs, preventing uptake of calcium, leading to hypercalcemia-induced seizures. Neuronal excitation is similarly calcium-dependent, and as such, calcium disturbances can lead to neurological disturbances [19, 20]. Hypercalcemia, when mild or moderate, is more associated with neurologic depression characterized by fatigue, muscle weakness, and confusion [19, 20]. However, a paradoxical relationship exists when severe hypercalcemia can lead to cerebral vasoconstriction, impaired neurotransmission, and disrupted cerebral metabolism [19, 20]. Thus, the compounded hypercalcemia caused by CCBs with MM could be responsible for the increased rate of seizure development. 

Dihydropyridines vs Non-dihydropyridines

Of the dihydropyridines studied, including amlodipine, nicardipine, and nifedipine, there appeared to be a consistent trend. Cohorts with MM and not on dihydropyridine CCBs demonstrated decreased risk of HD but increased risk of AF/AFL and VF/VT. Dihydropyridine CCBs are typically known for L-type voltage-gated calcium channel inhibition in vascular smooth muscle that reduces calcium influx and causes arteriolar smooth muscle relaxation [20-22]. They do not have as much of an effect on cardiac contractility or conduction but are associated with reflex tachycardia and occasionally hypotension [20-22]. It is interesting that this increased risk among MM patients for AF/AFL and VF/VT would be seen with dihydropyridine CCB cohorts and not compared to the cohorts taking non-dihydropyridine CCBs, which are more commonly used for cardiac conduction abnormalities. 

The lack of significant difference seen in rates of VF/VT among cohorts prescribed non-dihydropyridine CCBs, such as diltiazem or verapamil, further complicates this finding. Cohorts not taking non-dihydropyridine CCBs appeared to have a significantly reduced rate of AF/AFL development, which could be due to limitations of the study, despite efforts to control for them. It is also possible that even when patients required non-dihydropyridine CCBs for cardiac conditions existing prior to the index event, their risk for developing cardiac arrhythmias was worsened somehow by the MM diagnosis. This is possibly due to the increased risk of hypercalcemia-induced cardiac arrhythmias discussed above. Further research should aim to elucidate relationships touched upon by this study. 

It is also interesting to note that non-dihydropyridine CCBs appeared to provide a protective effect towards AKI and HD. Non-dihydropyridine CCBs have been shown to have renoprotective effects by reducing tubular injury from calcium build-up. Animal models have demonstrated that excess calcium influx into renal tubular cells, often seen in renal ischemic injury, is often responsible for tubular injury [21-23]. Non-dihydropyridine CCBs can mitigate this calcium influx, thus reducing rates of tubular injury [21-23]. With the hypercalcemia seen in MM, it is possible that the non-dihydropyridine CCBs were able to offer protection against this calcium influx. 

Limitations 

This study is limited by several factors, most notably its reliance on TriNetX queries of ICD-10 codes. Improper ICD-10 coding, whether due to inaccurately including or excluding a patient, can lead to skewed results. TriNetX also relies on HCOs to provide data according to the ICD-10 code queries sent to them. This reliance introduces an additional area of possible error; if HCOs do not respond, this can skew the data as well. Another limitation is our propensity-score match. Despite attempting to control for confounding variables by matching cohorts according to the conditions listed, it is possible that cohorts were still inadequately matched, leading to falsely significant differences. 

Another limitation is our inability to further stratify based on the degree of hypercalcemia experienced by our cohorts. A smaller study could potentially follow patients for a similar period and more closely assess how the degree of hypercalcemia is affected by CCBs as studied here. It is also possible that the degree of hypercalcemia could impact these outcomes as well, and further studies should focus on this.

## Conclusions

In conclusion, patients with MM without CCBs appear to be at a significantly reduced risk of developing AKIs, needing HD, seizures, AF/AFL, and VF/VT. Some mechanism-specific trends appear to exist, though they should be further explored. Dihydropyridine CCBs appear to increase the risk of cardiac arrhythmia in patients with MM, while non-dihydropyridine CCBs appear to protect patients with MM against the development of AKI and HD requirements. It is theorized that these findings are all related to MM-induced hypercalcemia, but further studies should explore all possible mechanisms. 
